# Burden of Chronic Cough and Refractory/Unexplained Chronic Cough in South Korea: A Multicenter, Observational Study (CHORUS)

**DOI:** 10.1007/s00408-026-00892-0

**Published:** 2026-05-08

**Authors:** Ji-Su Shim, Min-Hye Kim, Sung-Yoon Kang, Ji-Ho Lee, Eun-Jung Jo, Byung-Jae Lee, Sang-Heon Cho, JeeEun Jang, Woo-Jung Song

**Affiliations:** 1 Department of Internal Medicine, College of Medicine, Ewha Womans University, Seoul, South Korea; 2 Department of Internal Medicine, Gachon University Gil Medical Center, Incheon, South Korea; 3 Department of Internal Medicine, Yonsei University Wonju College of Medicine, Wonju, South Korea; 4 Department of Internal Medicine, Pusan National University College of Medicine, Busan, South Korea; 5 Division of Allergy, Department of Medicine, Samsung Medical Center, Sungkyunkwan University School of Medicine, Seoul, South Korea; 6 Division of Allergy and Clinical Immunology, Department of Internal Medicine, Seoul National University College of Medicine, Seoul, South Korea; 7 MSD Korea Ltd., Seoul, South Korea; 8 Department of Allergy and Clinical Immunology, Asan Medical Center, University of Ulsan College of Medicine, 88, Olympic-ro 43-gil, Songpa-gu, Seoul, 05505 Republic of Korea

**Keywords:** Chronic cough, Refractory cough, Unexplained cough, Disease burden

## Abstract

**Purpose:**

Refractory or unexplained chronic cough (RUCC) represents an unmet clinical need. This study aimed to explore the components constituting the disease burden in patients with RUCC by comparing clinical features, healthcare use, and patient-reported outcomes with those of chronic cough (CC) of recent onset (< 1 year).

**Methods:**

A total of 200 individuals were prospectively enrolled from seven referral clinics in South Korea, including 100 with RUCC and 100 with CC of recent onset (< 1 year). Data were obtained through structured interviews and medical record reviews. Measures included general and cough-specific quality-of-life, mood, cough hypersensitivity symptoms, work productivity, and healthcare utilization.

**Results:**

Individuals with RUCC were significantly older than those with CC (57.5 ± 15.1 vs. 48.9 ± 17.6 years) and had markedly longer cough duration (median 80 vs. 4 months). The RUCC group demonstrated higher out-of-pocket costs, more healthcare facility visits, and greater numbers of diagnostic tests and prescribed medications. However, cough-specific and general health-related quality of life scores showed no significant differences. Correlation analysis revealed comparable but stronger associations between cough-specific quality-of-life and general health outcomes in RUCC than in CC.

**Conclusion:**

RUCC imposes a substantial and multidimensional burden that extends beyond symptom severity. Chronicity, healthcare complexity, and psychosocial impact appear to be central features. Future evaluation tools should incorporate time and lived experience to more accurately capture the true burden of chronic cough.

**Supplementary Information:**

The online version contains supplementary material available at 10.1007/s00408-026-00892-0.

## Introduction

Refractory or unexplained chronic cough (RUCC) represents an unmet clinical need [1]. It refers to cases of chronic cough (CC) that remain refractory to etiological treatment. The prevalence of RUCC varies depending on the clinical referral settings, but data from referral clinics have reported prevalence rates of up to 60% [2–6].

The drugs currently recommended in the guidelines for the management of RUCC, including opiates, gabapentin, pregabalin, or amitriptyline [1, 7, 8], represent effective therapeutic options for selected patients. Some of these agents were originally developed for pain or neuropathic conditions and later repurposed for cough management, reflecting shared neural mechanisms relevant to RUCC [1, 9]. The antitussive efficacy of some opiates and neuromodulatory agents has been demonstrated in randomized controlled trials (RCTs); however, treatment response varies between individuals, and their use is often limited by adverse effects and concerns regarding long-term tolerability [4, 10–12]. Gefapixant, a first-in-class P2X3 receptor antagonist [13], has been licensed for the treatment of adults with RUCC in some countries in Europe and Japan [14], with real-world evidence supporting its efficacy, although its reimbursement and access remain limited in many countries. Thus, despite recent advances, important unmet needs remain.

Several metrics are used to measure the burden of a disease, including prevalence, incidence, cost, and impact on quality of life (QoL). CC is a prevalent condition and has a substantial impact on QoL [15–21]. RUCC is a chronic condition that often persists for several years or longer, with cough severity and frequency fluctuating over time [22–24]. In addition, patients with RUCC frequently face a range of challenges during their healthcare journey, including frustration, disappointment, perceived neglect, and overexposure to medications [25–29]. However, the overall disease burden of CC, and particularly that of RUCC, remains incompletely characterized.

In this study, we aimed to explore the components constituting the disease burden in patients with RUCC. For this purpose, we recruited patients with RUCC and compared their clinical characteristics, healthcare journeys, and QoL metrics with those of patients with CC of a recent onset (< 1 year).

## Materials and Methods

### Study Population

This multicenter observational study prospectively enrolled patients with RUCC or CC of recent onset (< 1 year) from seven referral clinics in South Korea. We enrolled patients with RUCC, who had cough for more than 1 year in duration and were receiving care with a diagnosis of refractory chronic cough (RCC; defined as a cough that persisted in patients with a comorbid condition associated with cough, such as asthma, rhinosinusitis, or gastroesophageal reflux disease [GERD], despite appropriate specific therapy) or unexplained chronic cough (UCC; defined as a persistent cough in patients without any comorbid conditions related to cough) despite guideline-based evaluation and treatment [1, 7]. For comparison, we also recruited those who had current cough persistent for 2–12 months in duration without any signs/symptoms that indicated a specific etiology (referred as CC group); study participants in the CC group had not yet fulfilled criteria for RCC or UCC at the time of enrollment. Patients were excluded if they had any medical condition that might increase the risk associated with participation or which might interfere with the study results, or if they had participated in a clinical trial involving an investigational agent within the previous 12 months. The study protocols were approved by the Institutional Review Boards of all participating institutions. All participants provided written informed consent and patient anonymity was preserved.

## Parameters

Data were collected from prospective patient interview and retrospective medical chart review during a routine care visit. No therapeutic intervention was given for the study purpose. Demographics, clinical characteristics, medical resource use and the patient’s healthcare journey were evaluated. Medical charts were reviewed for the period the patient had been treated for cough at the current hospital to obtain information on demographics, clinical characteristics, diagnostic tests, and medication prescription. Patients were interviewed to obtain information about healthcare utilization for cough prior to visiting the current clinics, including cough history, and health care resource utilization (HCRU).

Patient-reported outcomes (PROs) were measured to characterize the patient’s cough and health status: cough severity (100 mm Visual Analog Scale, VAS); general health-related QoL (EuroQol-5 Dimension [EQ-5D] [30], cough-specific QoL (Leicester Cough Questionnaire, LCQ) [17, 31], Hospital Anxiety and Depression Scale [HADS] [32]; cough-related throat sensations and cough triggers (Cough Hypersensitivity Questionnaire, CHQ) [33, 34]; and work productivity and non-work activities (Work Productivity and Activity Impairment, WPAI) [35].

The following variables were analyzed: history and clinical characteristics of cough (disease duration, subjective cough frequency and severity, cough hypersensitivity symptoms [CHQ], cough-related complications, and comorbidity), HCRU and treatment (type of cough-related visits, diagnostic tests, and treatment), and health outcomes. out-of-pocket costs were estimated based on patients’ self-reports, reflecting out-of-pocket expenditures, including hospital visits, prescription medications, and diagnostic tests incurred since the first occurrence of CC.

### Statistical Analysis

This was an exploratory and hypothesis-generating study in nature and no formal sample size calculation was performed. The target study size was selected empirically as 200 patients (100 with CC and 100 with RUCC). The analysis primarily compared patients with CC versus those with RUCC. Values were expressed as means ± standard deviations, medians (interquartile ranges [IQR]), or number with its proportions, and a *P* value of < 0.05 was considered statistically significant. Differences between groups (CC versus RUCC) were analyzed using the two-sample t-test or Mann Whitney U-test for continuous variables, and Chi-square test or Fisher’s exact test for categorical variables. The analysis of correlation between variables was conducted using Pearson’s correlation test. All statistical analyses were performed using SPSS version 22.0 (IBM, Armonk, NY, USA).

## Results

### Baseline Characteristics

A total of 200 patients were enrolled, of which 100 had recent-onset CC and 100 had RUCC (61 RCC and 39 UCC). Their baseline characteristics are presented in Table 1. Compared to subjects with CC, those with RUCC were significantly older (57.5 ± 15.1 vs. 48.9 ± 17.6 years, *P* < 0.001), but female was similarly predominant in both groups (74.0% vs. 67.0%, *P* = 0.278). There was no significant difference in BMI and family history of CC.

**Table 1:** Demographic characteristics of study participants

Variables	CC (*N* = 100)	RUCC (*N* = 100)	*P*-value
Age (years)	48.9 ± 17.6	57.5 ± 15.1	< 0.001
Female, %	67%	74%	0.278
BMI (kg/m^2^)	24.4 ± 4.7	24.4 ± 3.6	0.989
Smoking history, %			0.052
Current smoker	6%	3%	
Ex-smoker	22%	11%	
Never smoker	72%	86%	
Family history of CC, %	29%	29%	0.524
Duration of cough (months)	4.0 [3.0, 7.0]	80 [36.0, 149.5]	< 0.001
Cough-related complications, %			
Headache	13.0%	5.0%	0.048
Chest pain	9.0%	18.0%	0.063
Fatigue	13.0%	8.0%	0.249
Vomiting	1.0%	1.0%	1
Urinary incontinence	5.0%	11.0%	0.118
Sleep disturbance, %			
No	41.0%	60.0%	0.020
Mild	14.0%	16.0%	
Average	27.0%	11.0%	
Severe	12.0%	10.0%	
Extremely severe	6.0%	3.0%	
Comorbidity, %			
Diabetes mellitus	2.0%	2.0%	1
Hypertension	3.0%	7.0%	0.331
Dyslipidemia	7.0%	4.0%	0.537
Malignancy	3.0%	3.0%	1
Thyroid disease	5.0%	10.0%	0.179
Obstructive sleep apnea	2.0%	4.0%	0.683
Asthma	1.0%	17.0%	< 0.001
Allergic rhinitis	7.0%	30.0%	< 0.001
Chronic rhinosinusitis	4.0%	11.0%	0.105
Gastroesophageal reflux disease	6.0%	12.0%	0.138

Data are presented as number (%), mean ± standard deviation, or medians [interquartile ranges]

BMI: body mass index; CC: chronic cough; HCRU: healthcare resource utilization at facilities prior to the current hospital

Subjects with RUCC had a long duration of cough, which was significantly longer than those with CC (median 80 [IQR 36.0, 149.5] months vs. 4.0 [3.0, 7.0] months, *P* < 0.001). Subjects with RUCC reported more urinary incontinence (11.0% vs. 5.0%, *P* = 0.118) but less sleep disturbance (41.0% vs. 60.0%, *P* = 0.020). However, there were no significant differences in PRO scores: cough severity VAS, LCQ, CHQ, EQ-5D index, HADS, and WPAI (Table 2).

**Table 2:** Patient-reported outcomes of study participants

Variables	CC (*N* = 100)	RUCC (*N* = 100)	*P*-value
Cough severity VAS	47.3 ± 21.1	45.5 ± 22.3	0.547
LCQ score	12.8 ± 3.9	13.5 ± 3.9	0.172
CHQ score	10.2 ± 3.6	10.7 ± 4.3	0.360
HADS depression	6.8 ± 4.1	6.5 ± 4.4	0.693
HADS anxiety	5.6 ± 4.1	5.2 ± 4.1	0.470
WPAI			
Absenteeism	3.7 ± 12.9 (*N* = 47)	4.3 ± 10.2 (*N* = 40)	0.821
Presenteeism	28.1 ± 25.2 (*N* = 48)	29.3 ± 28.4 (*N* = 41)	0.841
Total work productivity impairment	29.8 ± 27.1 (*N* = 48)	31.5 ± 29.2 (*N* = 41)	0.783
Total activity impairment	39.6 ± 26.3	36.3 ± 27.0	0.382
EQ-5D index	0.89 ± 0.08	0.88 ± 0.08	0.561

Data are presented as number (%), mean ± standard deviation, or medians [interquartile ranges]

CC, chronic cough; CHQ, Cough Hypersensitivity Questionnaire (range 0–22; higher scores indicate more symptoms related to cough triggers and throat sensations); EQ-5D, EuroQol-5 Dimension; HADS, Hospital Anxiety and Depression Scale; LCQ, Leicester Cough Questionnaire (range 3–21; lower scores indicate poorer cough-related quality of life); RUCC, refractory or unexplained chronic cough; VAS, Visual Analog Scale for cough severity (range 0–100; higher scores indicate greater cough severity); WPAI, Work Productivity and Activity Impairment

### Healthcare Utilization

Subjects with RUCC reported more HCRU prior to attending current hospitals (Table 3): compared to those with CC, subjects with RUCC reported significantly higher out-of-pocket costs due to cough (746.6 [373.3, 2,239.6] USD vs. 224.1 [74.7, 343.7] USD, *P* < 0.001), more history of previous tertiary HCRU (25.9% vs. 10.1% *P* = 0.007), as well as visits to general practitioners (75.3% vs. 39.6%, *P* = 0.027) and to allergists among specialties (11.8% vs. 0%, *P* = 0.001). The number of previous HCRU facilities (4.5 ± 9.7 vs. 2.1 ± 1.2, *P* = 0.013) and the number of such visits were also significantly higher in RUCC (34.8 ± 66.9 vs. 7.5 ± 8.1, *P* < 0.001).

**Table 3:** Differences in healthcare burden between patients with CC and those with RUCC

	CC (*N* = 100)	RUCC (*N* = 100)	*P*-value
Out-of-pocket cost due to cough (USD)	224.1 [74.7, 343.7	746.6 [373.3, 2239.6]	< 0.001
Number of current medications	3.8 ± 1.5	5.7 ± 2.8	< 0.001
Number of diagnostic tests conducted at current clinic	5.4 ± 2.6	6.6 ± 2.5	0.002
Previous HCRU, n (%)	89 (89.0%)	85 (85.0%)	0.915
Primary clinic, n (%)	78 (87.6%)	79 (92.0%)	0.239
Secondary clinic, n (%)	14 (15.7%)	19 (22.4%)	0.265
Tertiary clinic, n (%)	9 (10.1%)	22 (25.9%)	0.007
Number of HCRU facilities	2.1 ± 1.3 (*N* = 89)	4.5 ± 9.7 (*N* = 85)	0.013
Number of HCRU facility visits	7.5 ± 8.1 (*N* = 89)	34.8 ± 66.9 (*N* = 85)	< 0.001

Data are presented as number (%), mean ± standard deviation, or medians [interquartile ranges]

CC: chronic cough; RUCC: refractory or unexplained chronic cough; HCRU: health care resource utilization

At current clinics, subjects with RUCC also reported a higher number of prescribed medications (5.7 ± 2.8 vs. 3.8 ± 1.5, *P* < 0.001) and more diagnostic tests (6.6 ± 2.5 vs. 5.4 ± 2.6, *P* = 0.002; Table 3). The following cough-related medications were more frequently prescribed to patients with RUCC: inhaled corticosteroids–long-acting beta-2 agonists (ICS–LABA; 35.9% vs. 12.7%, *P* = 0.001), short-acting beta-2 agonists (SABA; 7.6% vs. 0%, *P* = 0.042), leukotriene receptor antagonists (LTRA; 60.9% vs. 39.7%, *P* = 0.009), intranasal corticosteroids (INS; 23.9% vs. 4.8%, *P* = 0.001), proton pump inhibitors (PPI; 41.3% vs. 17.5%, *P* = 0.002), and gabapentin (12.0% vs. 0%, *P* = 0.004).

While codeine prescriptions were more common in patients with RUCC (tablet: 63.0%, syrup: 51.5%) than in those with CC (tablet: 50.8%, syrup: 36.5%), these differences did not reach statistical significance (*P* > 0.05). The prescription rates of oral corticosteroids and macrolides were comparable between the two groups (approximately 30% and 13%, respectively; Table 4).

**Table 4:** Cough-related medications prescribed at the current hospital in patients with CC and RUCC

Macrolides	CC (*N* = 63)	RUCC (*N* = 92)	*P*-value
	8 (12.7%)	14 (15.2%)	0.659
Other antibiotics	2 (3.2%)	6 (6.5%)	0.355
Anti-histamines	39 (61.9%)	70 (76.1%)	0.058
Mucolytics	17 (27.0%)	23 (25.0%)	0.782
Pseudoephedrine	8 (12.7%)	20 (21.7%)	0.151
Codeine (tablet)	32 (50.8%)	58 (63.0%)	0.129
Codeine-containing syrup	23 (36.5%)	47 (51.1%)	0.073
ICS	7 (11.1%)	17 (18.5%)	0.213
ICS-LABA	8 (12.7%)	33 (35.9%)	0.001
Short-acting beta-2 agonist	0 (0%)	7 (7.6%)	0.042
Oral corticosteroids	19 (30.2%)	30 (32.6%)	0.747
Leukotriene receptor antagonists	25 (39.7%)	56 (60.9%)	0.009
Intranasal corticosteroids	3 (4.8%)	22 (23.9%)	0.001
Proton pump inhibitors	11 (17.5%)	38 (41.3%)	0.002
Other anti-tussive	32 (50.8%)	57 (62.0%)	0.167
Neuromodulator, any	10 (15.9%)	21 (22.8%)	0.288
Amitriptyline	10 (15.9%)	17 (18.5%)	0.674
Nortriptyline	0	2 (2.2%)	0.514
Gabapentin	0	11 (12.0%)	0.004
Pregabalin	0	1 (1.1%)	1

Data are presented as number (%)

CC: chronic cough; ICS: inhaled corticosteroids; LABA: long-acting bronchodilator; RUCC: refractory or unexplained chronic cough

Overall, more various diagnostic tests were conducted in patients with RUCC than in those with CC. Compared to the CC group, patients with RUCC had undergone significantly more diagnostic tests both at previous and current clinics. At previous clinics, RUCC patients were more likely to have received chest computed tomography (23 vs. 9, *P* = 0.007), bronchodilator testing (11 vs. 2, *P* = 0.010), and serum total IgE testing (4 vs. 0, *P* = 0.043). At current clinics, RUCC patients more frequently underwent chest X-ray (94 vs. 81, *P* = 0.005), induced sputum analysis (39 vs. 19, *P* = 0.002), nasal endoscopy (18 vs. 4, *P* = 0.002), and allergen skin prick testing (34 vs. 15, *P* = 0.002).

Other tests, including spirometry, FeNO, sinus imaging, and various blood tests, were more commonly performed in the RUCC group but did not show statistically significant differences (Supplementary Table S1).

## Correlation Between Cough-Specific and General Health PRO Scores

In both CC and RUCC, LCQ scores, a measure of cough-specific QoL, showed significant correlations with health-related PRO scores, including HADS, WPAI, and EQ-5D index, with the degrees of correlations being generally stronger in RUCC. CHQ score, a measure of cough-related throat sensations and cough triggers, significantly correlated with HADS and WPAI scores in subjects with RUCC, but not in those with CC (Table 5).

**Table 5:** Correlation analysis of cough-specific and general health-related patient-reported outcome measures in CC and RUCC groups

		HADS				WPAI								EuroQoL	
		Depression	*P*-value	Anxiety	*P*-value	Presenteeism	*P*-value	Absenteeism	*P*-value	Total activity impairment	*P*-value	Total work productivity impairment	*P*-value	EQ-5D index	*P*-value
CC	CHQ	0.10	0.334	0.11	0.268	0.14	0.338	− 0.05	0.756	0.22	0.025	0.12	0.426	− 0.24	0.015
	LCQ	− 0.35	< 0.001	− 0.43	< 0.001	− 0.48	< 0.001	− 0.001	0.994	− 0.59	< 0.001	− 0.44	0.002	0.48	< 0.001
RUCC	CHQ	0.40	< 0.001	0.40	< 0.001	0.36	0.022	0.21	0.189	0.38	< 0.001	0.39	0.013	− 0.39	< 0.001
	LCQ	− 0.49	< 0.001	− 0.45	< 0.001	− 0.63	< 0.001	− 0.03	0.870	− 0.53	< 0.001	− 0.58	< 0.001	0.47	< 0.001

Data are presented as Pearson r coefficients and *p* values

## Discussion

This multicenter observational study provides a comprehensive characterization of the disease burden associated with RUCC, in comparison with less prolonged cases of CC. Our findings highlight the multifaceted burden of RUCC in terms of disease duration, healthcare utilization, treatment patterns, certain cough complications such as urinary incontinence, and PROs, thereby underscoring the clinical and societal impact of this condition.

First, we found that patients with RUCC had a markedly longer duration of cough symptoms and reported greater healthcare utilization across multiple domains. These included higher numbers of healthcare facility visits, broader diagnostic workups, and greater out-of-pocket medical expenditures. The higher number of diagnostic tests performed at the current clinics in patients with RUCC likely reflects the need to verify previous diagnostic findings and to re-evaluate the presence of potentially overlooked treatable traits. Importantly, even prior to visiting the current clinics, patients with RUCC had already undergone a number of diagnostic evaluations and visited multiple providers over a prolonged healthcare journey, often without achieving meaningful and sustained cough control. This trajectory reflects the complex and often fragmented care pathway typical of patients with RUCC and aligns with previous reports describing the diagnostic and therapeutic challenges [24, 27, 36–38].

Second, RUCC was associated with increased use of pharmacologic therapies. Compared to the CC group, patients with RUCC were more likely to receive multiple medications, including ICS–LABA combinations, LTRA, INS, and PPI. While these findings reflect the empiric and often trial-and-error nature of current management strategies for RUCC, it is notable that the use of neuromodulators and codeine, despite being higher in RUCC, did not significantly differ between groups. This suggests both a limitation in treatment effectiveness and a potential ceiling in real-world prescribing practices, consistent with earlier observations that currently available antitussives offer only modest benefit and are often accompanied by adverse effect [1, 39].

Third, although RUCC patients experienced more severe and longstanding cough-related complications, such as urinary incontinence, their scores on cough-specific and general health-related PROs were not significantly worse than those of the CC group. This may reflect a degree of psychological adaptation over time, or it may point to limitations in the sensitivity of PRO instruments to fully capture the fluctuating nature of lived experience of chronic cough. Interestingly, correlation analysis revealed stronger associations between cough-specific QoL (LCQ) and general health outcomes (EQ-5D, HADS, WPAI) in RUCC than in CC, suggesting that cough symptoms in RUCC may have more pervasive effects on emotional well-being, daily functioning, and perceived health status. In addition, CHQ scores correlated with anxiety and work productivity impairment in the RUCC group, but not in CC, supporting the notion that hypersensitivity-related sensory disturbances play a more central role in patients with longstanding refractory cough [40]. This also reinforces conceptual frameworks that position RUCC as a disorder of peripheral and central neural dysregulation, requiring mechanism-targeted approaches beyond empiric treatment [1, 41–44].

Based on the findings of this study, we believe that evaluating the disease burden of RUCC and CC requires a multidimensional approach rather than relying on a single measurement tool. Although it is well established that chronic cough has a substantial impact on QoL [16–20, 45, 46], and although generic health utility measures such as the EQ-5D or SF-36 are often used to estimate quality-adjusted life years, these instruments have important limitations. Their short recall periods and limited sensitivity to day-to-day variability make them less suitable for capturing the fluctuating nature of chronic cough [22, 47, 48]. In our study, EQ-5D index scores for both RUCC and CC appeared higher than those reported in previous domestic and international studies [16, 20, 46], suggesting that these tools may underestimate the actual burden, particularly if measured at a single time point.

In this context, we speculate that a more comprehensive assessment should include various direct and indirect indicators, such as healthcare costs, functional impairment, and patient experiences during the diagnostic and treatment process, including frustration, uncertainty, and dissatisfaction. In particular, we consider that one of the most important contributors to the disease burden in RUCC is the prolonged duration of illness. Our data showed a median duration of 80 months, and prior studies have reported durations often exceeding 10 years [13, 49]. When this prolonged time course is combined with the lack of an established diagnosis or effective therapy, it can significantly amplify the burden patients experience.

The passage of time itself becomes a critical factor in disease burden estimation. However, no current tool quantitatively incorporates time as a variable when measuring the disease burden of chronic cough. Developing such instruments may be essential for fully capturing the cumulative burden associated with chronic, undiagnosed, and treatment-resistant cough.

In the present study, we included a comparator group of patients with recent-onset CC without clinical features suggesting a specific etiology to better contextualize the disease burden of RUCC. Notably, despite the marked difference in cough duration, the two groups exhibited largely similar clinical characteristics. Age differences between the groups closely paralleled the differences in cough duration, and cough-related profiles were broadly comparable. Although LCQ scores in RUCC showed slightly stronger correlations with general health-related PROs, and CHQ scores were more closely associated with anxiety and work productivity impairment in RUCC, the overall patterns remained similar between the groups. These findings are consistent with a recent RCT demonstrating that the efficacy of gefapixant in patients with recent-onset chronic cough (< 1 year) is comparable to that observed in patients with RCC/UCC [13, 50]. In addition, prior analyses from the Korean National Health and Nutrition Examination Survey have shown a bimodal distribution of cough duration in community-dwelling adults, with peaks at ≤ 2 months and ≥ 1 year [51].

Taken together, the marked clinical similarity between patients with recent-onset CC without a clearly identifiable etiology and those with longstanding RUCC supports the concept of chronic cough as a distinct disease entity [1, 9, 43, 44]. Recent-onset CC, particularly in the absence of evident etiological clues, may already share key pathophysiological features with RUCC or may carry a higher risk of progression to a refractory phenotype. Although the likelihood of spontaneous remission in the recent-onset CC group remains to be determined, our findings suggest that defining RCC/UCC by a duration threshold of ≥ 1 year may be suboptimal. Instead, persisting for more than 2 months without an identifiable underlying cause may already warrant evaluation and intervention for cough hypersensitivity as a potential treatable trait.

Several limitations should be commented. First, the observational nature of the study precludes causal inference. Second, while we included a control group with CC of shorter duration, heterogeneity in etiology and treatment history may still confound comparisons. In addition, because this study was cross-sectional and did not include longitudinal follow-up, we could not evaluate remission over time, which may be an important clinical distinction between recent-onset CC and established RUCC. Third, cost estimates were based on patient self-reports and reflected out-of-pocket expenditures only, which likely underestimated the true economic burden of RUCC. Costs borne by insurers or the healthcare system were not captured, and the estimates may also have been affected by recall bias, particularly given the long cough duration in the RUCC group. Furthermore, differences in healthcare systems and reimbursement policies across countries may also contribute to the observed cost differences. For example, a recent French study using the French National Health Insurance Database reported 12-month total costs of €3,878 for non-RCC and €5,159 for RCC patients [29]. Finally, most participants were recruited from tertiary clinics, which may limit the generalizability of findings to primary care or community settings.

In conclusion, this study highlights the multidimensional disease burden associated with RUCC. Beyond symptom severity, RUCC is characterized by prolonged illness duration, repeated healthcare use, polypharmacy, functional impairment, and emotional distress. The findings underscore the limitations of current assessment tools and support the need for a more holistic evaluation framework that includes both objective and patient-centered metrics. Importantly, time itself, which reflects the chronicity of cough and delay in achieving diagnostic or therapeutic clarity, emerges as a central yet underappreciated component of disease burden. As the field moves toward more mechanism-based treatment strategies, future research should also prioritize the development of validated tools that incorporate temporal and experiential dimensions to better quantify the real-life impact of chronic cough. Finally, the observed similarity between patients with recent-onset CC without an identifiable etiology and RUCC suggests a continuum rather than a dichotomy, with potential implications for earlier recognition and intervention.

## Supplementary Information

Below is the link to the electronic supplementary material.

Supplementary Material 1

## Data Availability

The datasets generated and/or analysed during the current study are not publicly available due to patient privacy restrictions but are available from the corresponding author on reasonable request.

## Abbreviations

CC: Chronic cough
CHQ: Cough hypersensitivity questionnaire
EQ-5D: EuroQol-5 dimension
HADS: Hospital anxiety and depression scale
ICS: Inhaled corticosteroids
INS: Intranasal corticosteroids
IQR: Interquartile ranges
LCQ: Leicester cough questionnaire
LTRA: Leukotriene receptor antagonists
LABA: Long-acting beta-2 agonist
PRO: Patient-reported outcomes
SABA: Short-acting beta-2 agonist
PPI: Proton pump inhibitors
QoL: Quality of life
RCC: Refractory CC
RUCC: Refractory or unexplained chronic cough
TAI: Total activity impairment
TWPI: Total work productivity impairment
VAS: Visual analog scale
UCC: Unexplained CC
WPAI: Work productivity and activity impairment
