# Supplementary material for: Burden of Chronic Cough and Refractory/Unexplained Chronic Cough in South Korea: A Multicenter, Observational Study (CHORUS)
Source: Lung. 2026 May 8;204(1):29. doi: 10.1007/s00408-026-00892-0 (PMC13156121; doi:10.1007/s00408-026-00892-0)
Supplement: Supplementary file 1 — Supplementary Material 1 [file 408_2026_892_MOESM1_ESM.docx]

**Supplementary Table S1. Comparison of diagnostic test use at previous vs current clinics in patients with CC and RUCC.**

|  | **At previous clinics** | |  | **At current clinic** | |  |
| --- | --- | --- | --- | --- | --- | --- |
|  | **CC** | **RUCC** | **P value** | **CC** | **RUCC** | **P value** |
| **Chest X-ray** | 54 | 59 | 0.476 | 81 | 94 | 0.005 |
| **Chest CT** | 9 | 23 | 0.007 | 22 | 30 | 0.197 |
| **Spirometry** | 16 | 27 | 0.058 | 49 | 52 | 0.671 |
| **Spirometry with bronchial challenge test** | 4 | 11 | 0.060 | 33 | 46 | 0.060 |
| **Spirometry with bronchodilator test** | 2 | 11 | 0.010 | 21 | 30 | 0.144 |
| **FeNO** | 8 | 13 | 0.194 | 65 | 73 | 0.221 |
| **CBC** | 3 | 7 | 0.707 | 52 | 59 | 0.319 |
| **Induced sputum** | 0 | 3 | 0.080 | 19 | 39 | 0.002 |
| **Sinus X-ray** | 16 | 22 | 0.280 | 55 | 68 | 0.059 |
| **Sinus CT** | 1 | 3 | 0.312 | 1 | 2 | 0.561 |
| **Nasal endoscopy** | 4 | 5 | 0.773 | 4 | 18 | 0.002 |
| **Laryngoscopy** | 5 | 2 | 0.248 | 3 | 6 | 0.306 |
| **Allergen skin prick test** | 6 | 13 | 0.091 | 15 | 34 | 0.002 |
| **Serum specific IgE test** | 3 | 6 | 0.306 | 4 | 7 | 0.352 |
| **Serum total IgE test** | 0 | 4 | 0.043 | 43 | 44 | 0.887 |
| **Sputum AFB stain** | 0 | 2 | 0.155 | 13 | 10 | 0.506 |
| **Bronchoscopy** | 0 | 1 | 0.316 | 0 | 1 | 0.316 |

Data are presented as number.

AFB, Acid-Fast Bacilli; CC, chronic cough; RUCC, refractory or unexplained chronic cough; CT, computed tomography; FeNO, fractional exhaled nitric oxide; CBC, complete blood count; IgE, immunoglobulin E; AFB, Acid-Fast Bacilli.
